# Validating archetypes for the Multiple Sclerosis Functional Composite

**DOI:** 10.1186/1472-6947-14-64

**Published:** 2014-08-03

**Authors:** Michael Braun, Alexander Ulrich Brandt, Stefan Schulz, Martin Boeker

**Affiliations:** 1 Center for Medical Biometry and Medical Informatics, Medical Center – University of Freiburg, Stefan-Meier-Str. 26, 79104 Freiburg, Germany; 2 NeuroCure Clinical Research Center, Charité – Universitätsmedizin Berlin, Charitéplatz 1, 10117 Berlin, Germany; 3 Motognosis UG, Debenzer Str. 73, 12683 Berlin, Germany; 4 Institute for Medical Informatics, Statistics and Documentation; Medical University of Graz, Auenbruggerplatz 2, 8036 Graz, Austria

**Keywords:** Electronic health records, Multiple sclerosis, Medical documentation, Information models, Archetypes

## Abstract

**Background:**

Numerous information models for electronic health records, such as openEHR archetypes are available. The quality of such clinical models is important to guarantee standardised semantics and to facilitate their interoperability. However, validation aspects are not regarded sufficiently yet. The objective of this report is to investigate the feasibility of archetype development and its community-based validation process, presuming that this review process is a practical way to ensure high-quality information models amending the formal reference model definitions.

**Methods:**

A standard archetype development approach was applied on a case set of three clinical tests for multiple sclerosis assessment: After an analysis of the tests, the obtained data elements were organised and structured. The appropriate archetype class was selected and the data elements were implemented in an iterative refinement process. Clinical and information modelling experts validated the models in a structured review process.

**Results:**

Four new archetypes were developed and publicly deployed in the openEHR Clinical Knowledge Manager, an online platform provided by the openEHR Foundation. Afterwards, these four archetypes were validated by domain experts in a team review. The review was a formalised process, organised in the Clinical Knowledge Manager. Both, development and review process turned out to be time-consuming tasks, mostly due to difficult selection processes between alternative modelling approaches. The archetype review was a straightforward team process with the goal to validate archetypes pragmatically.

**Conclusions:**

The quality of medical information models is crucial to guarantee standardised semantic representation in order to improve interoperability. The validation process is a practical way to better harmonise models that diverge due to necessary flexibility left open by the underlying formal reference model definitions.

This case study provides evidence that both community- and tool-enabled review processes, structured in the Clinical Knowledge Manager, ensure archetype quality. It offers a pragmatic but feasible way to reduce variation in the representation of clinical information models towards a more unified and interoperable model.

## Background

### Introduction

There is a desideratum that high-quality clinical information models should be key parts of electronic health records (EHRs) [1], as they form reusable semantic artefacts [2,3]. Several standards have been created to represent and exchange electronic health data [4-6]. Among the most prominent approaches there are Health Level Seven (HL7) [7], openEHR [8,9], and its subset the European and ISO Standard 13606 (Health informatics – Electronic health record communication) [10,11]. These standards meet two key requirements for EHRs: syntactic interoperability and semantic interpretability. The systems should interoperate on the data level, as well as on the level of intended clinical meaning [1]. Whereas HL7 CDA (Clinical Document Architecture) is a widely adopted standard to document clinical information, openEHR has advantages in modelling what had been termed clinical concepts [12,13]. The Clinical Information Modelling Initiative (CIMI) also uses the openEHR approach [14]. CIMI is an international collaboration dedicated to provide a common format for detailed specifications for the representation of semantically interoperable health information. In an openEHR context the term clinical concepts does not signify entities of meaning like in terminologies or ontologies, but structured sets of data items to be recorded in a clinical context, e.g. for a specific clinical situation such as blood pressure or body weight observation. Numerous openEHR models have been developed. Several hundreds of them are freely accessible throughout online repositories, such as the Clinical Knowledge Manager (CKM) [15], provided by the openEHR Foundation [16], a not-for-profit company founded by the University College London (UK) and Ocean Informatics (Australia).

OpenEHR utilises a modelling approach specifying the information required to document any given clinical situation as a computable expression of a domain content model, called archetype [17-20]. In ISO 13972, similar constructs are named Detailed Clinical Models. Archetypes are reusable formal clinical models. They are expressed as a computable set of constraints based on a reference model. Typical clinical situations to be documented using archetypes include blood glucose measurements, diagnosis, microbiology results, medication, and adverse reactions.

An archetype specifies all the information a clinician might want to report about a particular clinical scenario [21]. However, there is no formal or quantitative correctness or completeness measure for archetype *content.* Formal criteria, indicators or patterns upon which some sort of comparison could be based on are lacking. Even though a reasoning method for validation based on the Web Ontology Language (OWL) was proposed [14], which can help to detect inconsistencies, it is not possible to compute their *quality* automatically. Although archetype-based EHRs exchange formalised clinical data, “there is a risk that mistaken interpretations may lower the accuracy of the communicated record and negatively impact on the quality of medical care” [3]. Also, the quality of the interfaces derived from those models like archetypes may have a direct impact on the quality of data in the future clinical use [22,23]. Improved user-interface design can offer lower interface error rates [24]. However, most of the archetypes are still in draft status, which mean that their quality assessment, to be examined in a standardised review process provided by the openEHR Foundation, is still pending. Until now, only few archetypes have been internationally peer reviewed by domain experts and received *published* status (as a visible sign).

Not many publications focus on the development of archetypes [3]. In [25] the immaturity of the openEHR approach and the available modelling tools is criticised. It states that developing high-quality archetypes is challenging and complex, archetypes do sometimes overlap, and the search for appropriate archetypes is time-consuming. According to [26], the design of archetype systems is not trivial, but archetypes are suitable to solve the problem of EHR storage and interoperability.

### Objective

The objective of this work is to thoroughly describe a case report about the openEHR development and validation process. Of particular interest is hereby the modelling process, including a review to validate archetype content. This open community-based approach is a unique feature of openEHR and has not been described before in such detail.

Furthermore, we wanted to discuss whether the archetype team review process can improve the semantic quality of archetypes beyond what is formally defined by the underlying openEHR reference model. We anticipate the openEHR team review process as a feasible way for validating archetypes and augmenting their semantics.

Our scenario has been the Multiple Sclerosis Functional Composite (MSFC) [27], a performance scale for the assessment of Multiple Sclerosis (MS) patients consisting of three neurological tests. Of further interest was hereby the suitability of archetypes for representing complex heterogeneous clinical tests. In particular, we traversed the workflow for the development and validation of archetypes regarding its practicability.

### Background and significance

#### Background of the openEHR approach

It requires established standardised medical information model specifications to ensure interchangeability and reusability of EHRs. The openEHR Foundation provides such specifications in the form of archetypes [28,29]. The two-layered modelling approach of openEHR allows clinical information to be specified in distinct models, called archetypes [18]. They provide the building blocks of information systems: syntactic interoperability and semantic interpretability [30]

In this two-layered approach, a repository based on a stable reference model, the first layer (see paragraph below), contains just generic knowledge and business rules [31]. So-called clinical knowledge, i.e. specifications of what is to be recorded by the model, is stored separately in the Archetype Model, which constitutes the second layer (see second paragraph below). This improves the flexibility of resulting EHR systems, because changes in the clinical knowledge can be dealt with only by revising archetypes, without compromising the integrity of information in the reference model [31].

The *Reference Information Model* defines a stable set of building blocks (modelling patterns), upon which the clinical models (archetypes) are specified. It includes complex data types, information patterns (e.g. entities called data, qualifier, state) and structural parts (e.g. composition, entry, tree) [31]. The clinical models are defined by constraining the reference model [31]. The reference model is designed to be invariant in the long term, which minimises the need for schema and software updates [32].

The *Archetype* or *Knowledge Model* is the backbone of the openEHR architecture. An archetype “is the model (or pattern) for the capture of clinical information – a machine readable specification of how to store patient data using the openEHR Reference Model” [31]. Each archetype comprehensively describes the information to be collected in a certain clinical record. These discrete models are intended to be directly instantiated with patient data in the clinical information system [31].

There are three different categories of archetypes, each corresponding to classes in the openEHR reference model: Thematic archetypes of *Compositions*, which correspond to commonly used clinical documents. Organisational archetypes of *Sections* assist human navigation within EHRs. *Entries* are the most common and fundamental building blocks of EHRs. There are four different classes of these descriptive archetypes in the openEHR reference model to represent different kinds of clinical data: *Observation* (e.g. to record measurable or observable data), *Evaluation* (e.g. to record clinical findings), *Instruction* (e.g. to record the initiation of a workflow process), and *Action* (e.g. to record clinical activities) [31].

The content of archetypes should ideally be language-independent, so that they can be translated, interpreted, and viewed in another language without losing meaning or context [31]. This is ideally assured if their meaning-bearing elements are bound to a multilingual terminological standard like SNOMED CT, which provides a clear model of meaning by well-defined concepts and attached terms in different languages. Archetypes can be assembled and specialised to form compositions in the EHR for a specific clinical purpose, called templates [33]. These aggregations can form data sets corresponding to clinical tasks, such as antenatal exams or discharge summaries [9].

### Projects and applications utilising openEHR archetypes

There are numerous projects utilising openEHR archetypes. A literature review of archetype-based EHRs is available in [26]. It gives an overview about worldwide archetype use and archetypes for electronic decision support systems.

An editor that supports manual and semi-automatic creation of bindings between archetypes and terminology systems is presented in [34]. Not only finding correct terms is difficult [34]; another challenge is the boundary problem between archetypes and terminologies [26]: what should be represented in the archetype, what in the terminology?

Various EHR approaches, including HL7 and openEHR, are evaluated in [5] versus the Generic Component Model (GCM), a reference architecture for providing a multi-model approach to any system architecture. Reflecting all GCM paradigms is demanded as crucial. The formalisation of most of the approaches is criticised as “underdeveloped”. HL7 has the largest intersection with the GCM, but many specifications are still inconsistent. The ontology-driven architecture [35,36] of the openEHR approach is valued in particular. However, no meta-model was found in the openEHR system architecture [5].

The expression of clinical data sets (CDS) for paediatric oncology with openEHR archetypes was investigated in [37]. 48 archetypes were developed and it was stated that they are of better quality than original CDS (they came across CDS problems and openEHR-based solutions). The conclusion was that archetypes are a robust base for ubiquitous computing. The approach is feasible and semantically interoperable, but unable to overcome all barriers.

### Archetype modelling strategies

The formal language to express openEHR archetypes is the Archetype Definition Language (ADL). It provides syntax for describing constraints on information entities whose data is described by the reference model. Although there is a rich set of specifications for the modelling language itself and the general architecture available, not much information about the actual modelling process is given. A set of 14 general “Archetype Design Principles” [18] is available and a tutorial on building archetypes [38]. In addition, on the openEHR webpages certain information is available about how to choose the right class for the archetype and other frequently asked questions. In sum, this is not much source for developing high-quality archetypes, rather a starting point for beginners. Therefore, the development of a first archetype is not a straightforward task.

### Application domain: the Multiple Sclerosis Functional Composite

The MSFC is a standardised quantitative assessment instrument for clinical practice and research on Multiple Sclerosis (MS). The US National Multiple Sclerosis Society originally developed it for the application in clinical trials. An administration and scoring manual is publicly available [27].

The well-established and widely used MSFC consists of three separate neurological tests, which are important for the diagnosis of MS. The cognitive and motor skills of patients are assessed by the following tests:

1. The *Timed 25-Foot Walk* (T25FW) is a quantitative walking test of lower extremity function.

2. The *Nine Hole Peg Test* (NHPT), a quantitative measure of upper extremity function, is used for rapid assessment of a subjectâ€™s finger dexterity.

3. The *Paced Auditory Serial Addition Test* (PASAT) measures the cognitive function. It specifically assesses the auditory information processing speed and flexibility, as well as the calculation ability of patients.

The results of three separate tests are settled as a total value (the MSFC Score), which correlates with the severity of the disease with respect to a reference population. The average result of each test is individually computed to a Z-score and averaged in the MSFC Score. All three measures making up the MSFC showed to have good inter-rater and test-retest reliability [39].

## Methods

### Archetype modelling

The procedure models mentioned follow similar major steps, with varying levels of detail: At the beginning, an analysis of the data to be represented is performed. In the following step(s), the data obtained is organised and structured. After choosing the appropriate archetype class, the data fields (structure, meta-information, constraints etc.) are implemented. As archetypes are usually developed in a team, an iterative refinement process is suitable. The authoring group consisted of two physicians (one a clinical MS expert but with no modelling experience, the other one a medical informatics expert with no MS expertise but with modelling experience), two technicians and one modelling expert (although with no previous expertise in archetype modelling).

OpenEHR archetypes are very flexible and expressible models. The approach is open to deal with any possible scenario and for interoperability. It offers just a general methodology, leaving a lot of flexibility (regarding structure etc.) to the modellers. This means that for many design decisions there are no formal constraints, making some selections decisions difficult. Instead of narrowing the selection decisions down (and risking to lose expressiveness), they implemented for a governance review process to ensure consistency and quality of the resulting model (see corresponding section after the archetype development as well as in the discussion).

### Developing archetypes for the MSFC

**Figure 1:**
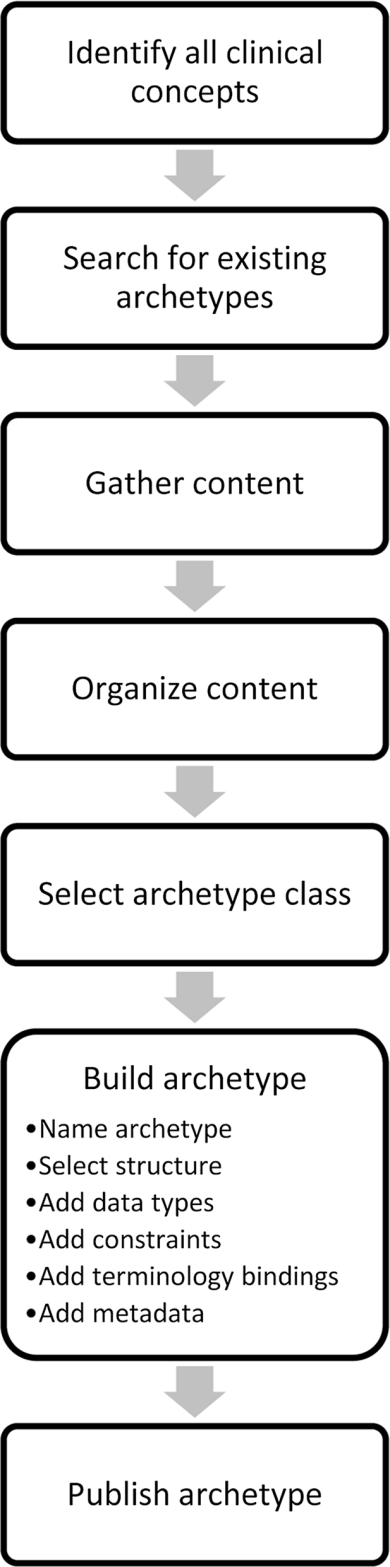
**Development steps for building a new archetype.** If no existing archetype can be reused, the shown steps should be performed, based on [38].

An individual archetype was developed for each of the three MSFC tests: *Timed 25-Foot Walk*, *Nine Hole Peg Test* and *Paced Auditory Serial Addition Test*. In addition, one archetype representing the *MSFC Score* was modelled. We used the current stable release ADL 1.4 [28]. The following methodology was used for each of the four archetypes, a modified approach of [38], see Figure 1:

At first, an analysis of the domain of discourse and the requirements was performed. The developer of an archetype should be deeply informed about the entity he wants to represent. Usually, it will be necessary to involve domain experts in the modelling process. In our case, this included a literature survey about each test, as well as an analysis of their record forms and administration instructions. One major principle of archetypes is that they should represent a maximum dataset [31,38] to maximise reuse and interoperability [35]. Therefore, all attributes clinicians might want to capture in a given scenario have to be identified.

Sometimes the instructions about how a test has to be administered are contradictory, like in the case of the Nine Hole Peg Test [27,40,41]. We always took the MSFC Manual as reference, but also covered variants of the test. Ambiguous topics, as well as test variants (necessary to maximise reuse) can lead to time-consuming modelling decisions.

As part of the analysis, a search for existing archetypes should be performed with the aim to reuse them. There are publicly accessible repositories such as the openEHR CKM. If no existing archetype can be adapted or reused, a new one has to be developed. We decided to design new archetypes, because we did not retrieve reusable archetypes on the internet or in openEHR repositories.

For each new archetype, the content was gathered, organised, and structured: The relevant items were identified from books, instruction manuals, record forms, and journal articles. Examples include the time needed to complete the 25-foot walk, the number of correct answers for addition test and confounding factors in all tests. The archetype header also includes a list of the references that were used to gather the content of the archetype, also demanded in [1]. After identifying the relevant content, organisation in mind maps is useful for further discussion including medical experts [3].

With the selection of the appropriate class of the archetype (the next step), the content was structured further, according to the specific class. Every class has a different structure and specific attributes for different parts of the clinical recording processes and workflow. As we record measurable data, the *Observation* class fits for all of the four MSFC archetypes. This class is structured into *Data*, *Events*, *Protocol,* and *State*. The Data part contains the core information (e.g. the MSFC score, the percentage of correct answers, or information when the trial was not completed and the reasons therefore). The Event part contains information about the timing of the observation. The Protocol part contains information on how the information was gathered or measured (e.g. if multiple attempts were needed to complete the task or which assistive devices were used). The State part contains information about the patient at the time when the information was collected, in our cases especially confounding factors for administering the tests. This information is important for the interpretation of the core information recorded in data.

After the selection of the appropriate class of the archetype, the actual implementation began: The archetype was named, structures were selected, and meta-information was given, including keywords, author, and contact information. Also required by [1] and already part of the ADL, copyright and version information were added automatically by the editing tool (see second paragraph below). Then we added the data elements with appropriate data types and descriptions. Occurrences and constraints were defined where necessary. Giving concise definitions of each concept, the purpose, use and misuse of the archetype, as well as setting appropriate constraints are challenging tasks that require a lot of coordination effort and expert opinion. This applies for both the development phase and on the review process.

In order to streamline the development process, we did not realise terminology bindings [34], i.e. connection to representational units in SNOMED CT, LOINC, ICD, or other terminology systems. We consider terminology bindings or, more generally, semantic annotations of archetype elements [42], as well as translations, as separate, non-trivial tasks. The binding of terminology codes is difficult to perform due to the large size of the terminologies [43]. Therefore, we focussed on the content review (next sections) and we will add terminology bindings in the future. We leave open whether this two-step approach is recommendable in all cases. There might be good reasons to perform terminology binding in an early step of archetype construction in order to prevent further misunderstandings by an unconscious choice of ambiguous terms.

During the whole process, we followed an iterative approach. We discussed the current state of development in regular intervals with medical experts and our project partners. A few times consensus was not reached during a meeting. Then further investigations were required to elaborate further on the benefits or weaknesses of different approaches. Usually, the questions were resolved in the following meeting. The general principle was to represent the existing record forms of the tests as precise as possible. We used the Archetype Editor as modelling tool, which is publicly available from openEHR and Ocean Informatics. This editor offers a graphical user interface that supports creating and editing openEHR archetypes on the client side. Authoring is supported by an intuitive drag-and-drop interface. Generated GUI mock-ups help users to visualise the meaning of the archetypes. Translations and bindings to terminologies can also be added using the Archetype Editor.

### Validation

Archetype design and validation can be time-consuming due to the lack of both domain expertise and modelling experience. There are two aspects of validation: syntactical and content validation. Technical aspects, such as syntax checking etc. can be covered easily by tools, like the Clinical Knowledge Manager or the ADL Workbench [44]. The four MSFC archetypes were syntactically validated by the ADL Workbench and the CKM. In addition, the models were checked and found to be correct by the online OWL-based reasoning tool Archeck, introduced in [14].

Furthermore, actual archetype content validation is crucial as well [23]. For example, there is not always a sharp separation in choosing the right data types or structuring content. It is also arguable what “right” means. Only clinical *domain* experts as well as *modelling* experts together can give advice and form consensus during review [45].

In order to upload, review, and publish the resulting four MSFC archetypes to the CKM, we contacted the leading editors of openEHR. Before the actual review process in the CKM (following section) we performed an informal internal check of the archetypes to meet certain (coding) conventions and had some improvements addressing their form, rather than modifying content.

### The team review process

The four MSFC archetypes were reviewed by international clinical and domain experts. The openEHR Archetype Team Review is a straightforward tool-based process in the CKM. This platform serves as archetype repository and revision control system, as well as a foundation for the review supporting and structuring the whole process. The review is based upon volunteers, who can participate after registration in the platform, affiliated or not affiliated with openEHR (not members of any openEHR committee) likewise. Registration is open to everyone who is interested in archetype usage, modelling, translation, and revision.

We applied the review process to the four archetypes, which is described as follows: The archetypes were initially uploaded where they automatically received *Draft* status. Then the process was initiated, and a review team was invited: Reviewers could be picked manually as well as via a search function, e.g. all CKM users with clinical expertise in Neurology and willing to volunteer in reviews were chosen. The team included reviewers affiliated and not affiliated with openEHR from universities, companies, and hospitals likewise. They received an invitation with a short description of the respective archetype, the CKM process, and a review checklist [46]. During the review, archetypes were flagged as *Team review*.

The review itself consisted of multiple iterations, called review rounds. Periods for the participation in and the completion of the review were given (typically two weeks). Reviewers could comment every aspect of the archetype. They should examine all items critically. This includes, but is not limited to concept names and descriptions, appropriate date types and structure, constraints and cardinalities, missing items and metadata. The archetype under review should also be checked for consistency of phrasing, expression, punctuation, and spelling. A comprehensive checklist is available in [46]. During this process, the reviewers can address questions and discuss every aspect of the model, like revising descriptions, adding record examples, clarifying meta-information, or adding constraints. The editors can also post specific questions to request expert opinion for certain modelling decisions.

To conclude the round, the reviewers gave an overall recommendation of the archetype (Accept, Minor Revision, Major Revision, Reject, or Abstain). At the end of each round, all comments were collected and a summary was written. The archetypes were checked out for changes and modified according to the reviewersâ€™ comments and suggestions. After performing the changes, the archetypes were uploaded again and a check-in into the CKM versioning system was done. The reviewers received a summary about the changes, and then the editors initiated the next review round. The models were refined with each round. When consensus was reached and all the reviewersâ€™ requests were met, the archetypes received published status.

## Results

In this work, we created four new archetypes representing the MSFC neurological test suite. All archetypes are available in English and German. They are of the *Observation* class, as they represent measurable test results. The archetypes were internationally reviewed by domain experts and published. All four models are publicly accessible on the internet in the CKM directly via the links below (without registration). They are available free of charge for organisational and individual use under Creative Commons Attribution-ShareAlike 3.0 Unported License (CC BY-SA [47], the same as e.g. Wikipedia):

• Timed 25-Foot Walk: http://www.openehr.org/ckm/#showArchetype_1013.1.1200

• Nine Hole Peg Test: http://www.openehr.org/ckm/#showArchetype_1013.1.1202

• Paced Auditory Serial Addition Test: http://www.openehr.org/ckm/#showArchetype_1013.1.1296

• MSFC Score: http://www.openehr.org/ckm/#showArchetype_1013.1.1368

**Figure 2:**
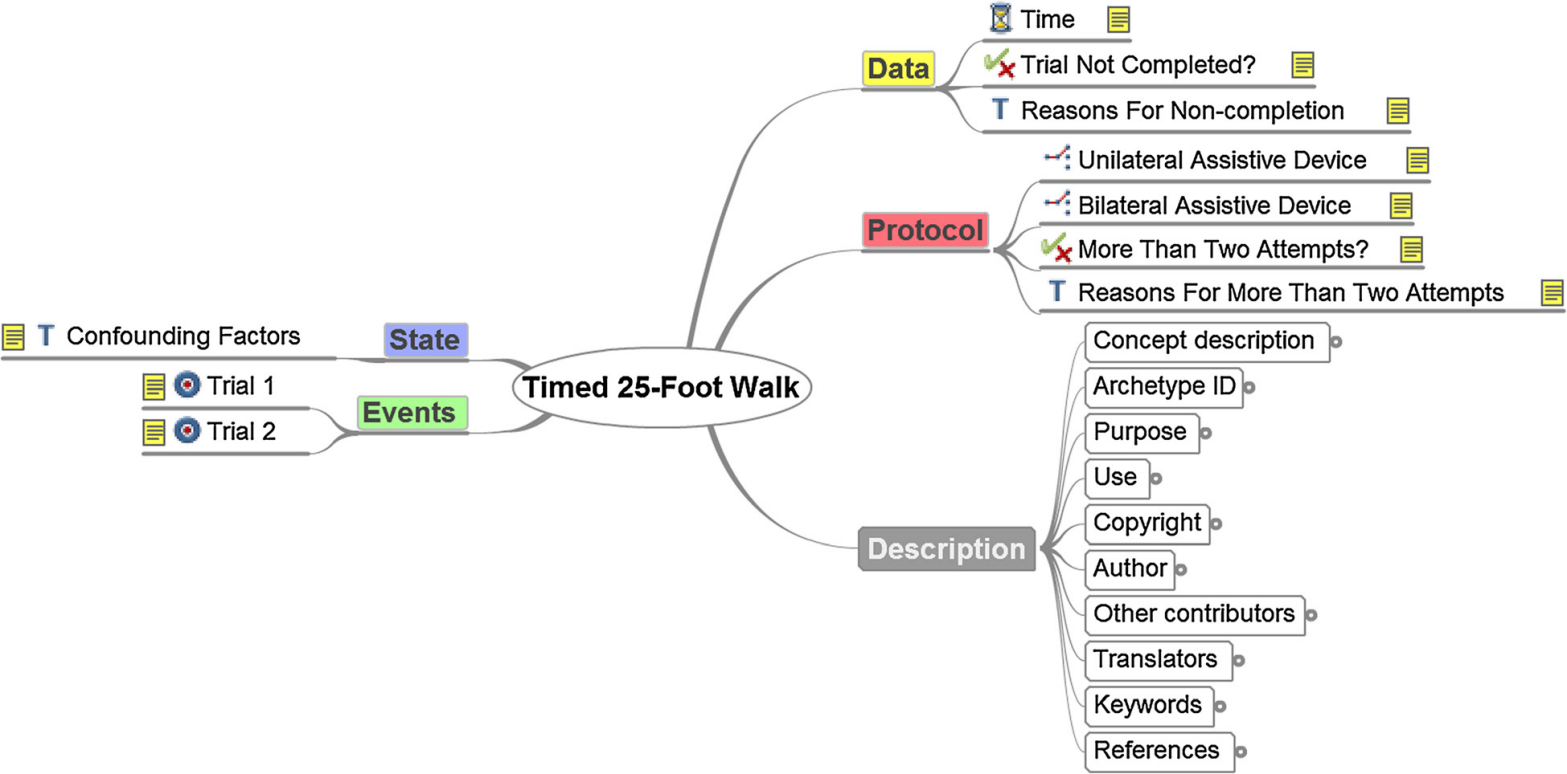
**The mind map representation of the Timed 25-Foot Walk archetype.** This figure is extracted from the CKM and shows the structure of the *Observation* class and the data elements of the archetype.

Figure 2 shows the mind map representation of the Timed 25-Foot Walk archetype. It illustrates the structure of the *Observation* class: Data, Events, Protocol and State, as well as Description for meta-information. This points out that there are many possibilities for structuring the data elements. All data elements and their type (e.g. Text, Boolean, Time) are visible but not their description or additional constraints.

The parts mentioned cannot always be differentiated precisely, especially between Data, State, and Protocol. In the beginning, it is not always clear whether a certain item (i.e. the dominant hand in the peg test) belongs to the core data that is observed, to the patientâ€™s state, or serves as additional information in the protocol. Even different medical experts might have different opinions. Therefore, this structuring process requires coordination effort with experts in the field, which can be time-consuming. This applies for both, the modelling phase and the later review process as well. Every detail of the archetypes can be seen in CKM via the links above. Therefore, we abstain to describe the resulting final models in details. CKM offers multiple sights on the models, like the mind map overview (as above), a detailed view of every concept description, and an ADL as well as an XML representation. The archetype history and review status is transparent online in the CKM.

Table 1 shows the metrics of the archetype review process, *viz.* how much feedback was received, and how many people were involved in the model refinement. The first line shows the number of data elements of each archetype. It did not change during the reviews in all four archetypes. The second line shows the number of review rounds needed until publication, followed by the number of single reviewers that participated, and the number of total reviews for each archetype. The fifth line shows the total number of comments given in all review rounds. From the reviewersâ€™ comments, we identified a number of change requests for the archetypes. Some of them occurred more than once, so the number of individual change requests is shown in the next line. The final line shows the overall number of individuals who contributed to the development and publication of each archetype.

**Table 1:** Metrics from the reviews of the four MSFC archetypes

**Archetype**	**Timed 25-Foot Walk**	**Nine Hole Peg Test**	**Paced Auditory Serial Addition Test**	**MSFC Score**
**Data elements**	20	28	25	17
**Review rounds**	3	2	1	1
**Reviewer**	8	5	4	2
**Reviews (total)**	14	8	4	2
**Comments (total)**	41	27	12	4
**Change requests**	42	29	13	4
**Individual requests**	31	26	11	4
**Changes implemented**	27	21	7	4
**Contributors**	11	10	11	8

We generally demonstrated that the openEHR approach is an adequate format for representing complex neurological tests, by modelling the three MSFC tests in detail as archetypes. However, the question arises to the expert quality and the validity of the models. Most, but not all of the reviewersâ€™ requests were met, mainly because of different understanding of the test instructions or alignment with modelling conventions. These issues were discussed and resolved online during the process. Most changes were of textual matter (clarifying descriptions, changing options, or giving examples) or adding/removing constraints. A more comprehensive survey, i.e. with the number of reviews in each round and the reviewersâ€™ recommendations can be seen online in CKM for each archetype, including its revision history. Single comments of the reviewers are visible only for editors and the other reviewers. Apart from that, the process is highly transparent.

Altogether, we proceeded sequentially and finished one archetype review before we initiated the next. Hence, the experiences from the previous reviews were incorporated in minor revisions of the following models. For example, some data elements (like confounding factors) appear in several archetypes. This may be a reason why later review rounds were shorter, because the quality of models was more elaborated at the beginning of the development process. The time from first review round initiation date to latest review round completion date took an average of 59.5 days (with a range 14–122 days). For the other eight archetypes that have received published status in CKM by then, the average period was even longer with 180 (40–323) days. These numbers intended as a rough guide making the interpretation difficult due to the small data pool. It is also difficult to generalise these numbers to different topics. The time needed depends in particular on the subject to be mapped, how structured and ambiguous it is, as well as on the team size and its experience. Further problems are the periods of idle time in the overall process. However, the CKM turned out to be a sophisticated collaborative platform, which helps to organise reviews as straightforward processes. On this basis, archetype refinement and content validation were conducted on a high-qualitative level.

## Discussion

In this work, we have presented four new archetypes for the MSFC. We have focussed on the development and validation process itself, which has not been described in detail before. The interface of the publicly available Archetype Editor supports the development process visually. However, the archetype development process was slowed down due to difficult selection decisions between alternative modelling approaches, also reported in [3]. All four archetypes were uploaded to the CKM, where they are publicly accessible. After validation by domain experts in the community-based review process, they received published status. Structured by the CKM, archetype review is a straightforward and well-organised team process with the goal to validate archetypes pragmatically. In this case report we provide evidence for the quality improvement of information by this community effort. We emphasise that content validation is crucial for quality enhancement and standardisation of openEHR information models, where a corresponding formal model must have its shortcomings to preserve the necessary flexibility.

Due to the lack of formal or quantitative correctness measurements for archetypes, the only way of assessing and improving the model quality is peer review by domain experts. This process ensures not only the quality of the models themselves, but also their acceptance by other clinical experts. Despite the wide agreement on the importance of the reviews (e.g. [45,48,49]), less than 5% of the CKM archetypes have been reviewed and received published status today. Significant further work needs to be done in order to ensure interoperable high-quality archetypes [1]. We hope that the presented work can lower the barrier for developers to request a formal review with the openEHR community. Only then, a sufficient foundation of validated archetypes will be publicly available and analyses with more numerous cases can be performed.

This also leads to the question whether the archetypes in draft state available in the CKM are “safe” to use or reuse (modelling step 2) in contrast to reviewed archetypes in published state. Before new archetypes are uploaded, they undergo an editorial appraisal to ensure a certain standard (mentioned earlier). CKM users are not permitted to upload new archetypes, only CKM editors can do this. Still, the quality of these archetypes may vary widely. They can be a starting point for review or may be in a state close to publication.

Once uploaded and online in the CKM, every user can comment any archetype and address e.g. missing aspects, different or country-specific views without formal review. The editors can then incorporate or discuss the change requests. But neither this nor the editorial check before upload can substitute a formal review by domain experts of the community. The audience is wider, as more people are involved and much more viewpoints are considered in the discussion.

A lot of archetypes are online for some years and some of them have received suggestions for improvement continuously. So they may have matured over time, even though the formal standard review is lacking. However, there may be indicators for the maturity of a draft archetype, like its history and revision, the number of contributors etc., but not a formal assessment. Based on the experience with our own archetypes, we advise to take a close look at archetypes in draft state. The potential user should be aware that the review could lead to significant changes. Hence, we do not recommend using draft archetypes in a clinical setting.

Our investigation of the modelling and validation process was affected by the domain of discourse. However, we do not believe that our setting (the MSFC) had an impact on the general illustration of the design and validation process (the steps performed are independent of the use case). The degree of formalisation and standardisation of the domain as well as of the material that is available as reference will indeed influence the process (e.g. more complex models may require more coordination effort).

Madsen et al. investigated sustainable clinical knowledge management with respect to the archetype development life cycle. A well-designed archetype for a given clinical entity should cover all of the data, independent of the use-case [35]. They proposed a process for archetype development with seven phases, related to the traditional software development lifecycle. The first phase is the planning phase, where the content is gathered from various sources and clinicians are engaged to ensure alignment with clinical requirements. The analysis phase includes data analysis and consolidation as well as inspection of existing archetypes. Following the requirement specification phase and archetype design phase, the fifth phase consists of test, evaluation, and review, concluded by the delivery and the maintenance phases.

To the authorsâ€™ knowledge, two more methodologies have been used for the design of archetypes: The AORTIS model [50] is a general scheme for summarising clinical information. It identifies five distinct stages: aggregation, organisation, reduction and/or transformation, interpretation, and synthesis of clinical data. Furthermore, [51] proposed the methodology “odma” (openEHR data modelling approach), also used in [3]. It consists of the five steps determining all items to be documented, merging these items into clinical concepts, matching the derived concepts against existing archetypes, developing archetypes, and designing templates. The work concludes that the two-layered modelling approach is a major advantage, but it is difficult and time-consuming to develop archetypes. Domain expertise is required. Further important development steps are (if not already integral part of the methodology): binding to external terminology systems, collaboration like reviewing and publishing the archetype, as well as adding the archetype to a template. Peer reviewed design and modification of archetypes is a prerequisite for high-quality models [49].

Although ADL is a sound and comprehensible formal basis for archetype development and integrated into tool support (e.g. CKM and Archetype Editor), we agree with other developers (e.g. [25]) that there is a shortage of modelling guidelines or best-practice recommendations. How to select from a multitude of potential equivalent models is not covered by the formal language definition. However, it remains unclear whether such modelling paradigms can even be described formally without the adverse effect of too limited modelling expressivity. This case study supports the development paradigm of combining a formal language definition with a regulated review process to achieve a pragmatic but feasible definition process of expressive, nevertheless standardised and interoperable information models.

Such a centralised and standardised review process can also help to come across ambiguous test instructions (as mentioned earlier), because the openEHR approach tries to involve clinicians from the beginning of the modelling process to ensure model consistency. Furthermore, model and style variations would probably occur even if best-practice recommendations and modelling guidelines were available, just because different designers may have different approaches. Again, archetype submission to CKM and centralised review will assure alignment with consistent modelling methodology [35].

### Limitations of the study

The results of this case report are not generalizable to all medical information modelling methodologies. They represent our personal experience from the development and review of the four MSFC archetypes. In the general topic of how to measure the quality of information models, we assessed the archetype review process as one way to improve the quality of the models. We do not know how other approaches (e.g. HL7) ensure high-quality models. Tools can cover syntactic correctness and consistency checks. Beyond that, there are (as far as we know) only non-quantitative indicators, such as expert opinions, the use in clinical application (as well as the quality of documentation), as well as links to terminologies (see next section). The assessment of all of these aspects needs to be done by medical experts. Therefore, it is essential to involve medical experts from the very beginning of the modelling process, as promoted by the openEHR approach.

The general idea of rating and commenting information models has also been implemented by Breil et al. [52] in a portal for Medical Data Models. A large number of record forms has been collected from various sources, and can also be imported from and exported to multiple formats. They do not provide a structured review but a simple five-star rating and comment function. This example shows that the idea of independent quality assessment by external experts (peer review) can be generalised to other applications outside the openEHR context. However, this may request significant work and additional research.

As far as we know, the four MSFC archetypes have not been used in a clinical situation. So an evaluation in clinical practice is still lacking. Recently, the archetype of the Nine Hole Peg Test has been translated into Chinese. Nevertheless, we think that we provide evidence for a method and feasibility of a structured development process for complex information models which results in reusable artefacts. Despite these major limitations, this work can add to a corpus of evidence on how to achieve a substantial amount of validated archetypes.

### Further research

A next step could be the binding to external terminologies like SNOMED CT, based on current work done by the CIMI and the SemanticHealthNet network [42]. Another important aspect is the discussion about quality criteria upon which the peer review should be based on. One criterion could be such a consequent terminology binding. This leads to the question of how to draw a line between content to be expressed by information models and content to be expressed by terminologies and ontologies, according to clear criteria [53,54].

A more thorough investigation of the lifecycle of information models (including their clinical application) should be done with respect to the efficiency of the review process: Are these models invariable for a long time and is therefore an expensive validation process justifiable? Even published archetypes may receive frequent change requests (due to new standards, treatments, regulatory, or terminological changes). It is also possible that different experts could have produced a slightly different outcome. Here, one should investigate if there could be determined a typical number of expert opinions to lead to a stable consent. However, archetypes represent current clinical practice. As such, published archetypes are not perfect, but they are likely to be the best and most flexible models available at present.

Furthermore, we found that some medical assessment instructions, e.g. how a certain test should be performed, are differing in literature. This leads to the question if a consensus forming process, like the team review for archetypes, could help to standardise medical procedures (bidirectional information flow).

## Conclusions

Assessing the quality of information models in a standardised way is an integral part of the development process. Several approaches to represent and exchange electronic health data are available. In a corpus of prior studies, a lot of effort has been spent for feasibility checks, to show that certain aspects can be represented. Now it is time to thoroughly investigate the quality of those models. Hereby, clinical experts and modelling expert have to work together in a process like the one we have illustrated.

Peer review is an important part of the development lifecycle for information models. It is crucial not only for the quality of the models, but also for the quality of the resulting applications and therefore patient safety. The development and validation process is time-consuming. However, the review can significantly improve the model quality, so we think it is worth the effort. During the whole development lifecycle, it is crucial to consult clinical experts, not only during the validation of the models. The CKM is a well-suited platform for international collaboration. Once a certain amount of stable models is available, fewer archetypes have to undergo design from scratch and the development times may shorten.

The openEHR approach offers great expressivity and flexibility. Although there is a shortage of modelling guidelines and best-practice recommendations, openEHR provides a community-driven validation process, which can partly fill the gap. In particular, the review process is a pragmatic way in achieving high-quality information models by integrating domain specialists.

## Competing interests

The authors declare that they have no competing interests.

## Authorsâ€™ contributions

The four authors are justifiably credited with authorship, according to the authorship criteria: StS, AUB, and MaB designed the study. MiB did the initial design of the archetypes and the final implementation. AUB and MaB contributed substantially to the archetype development process. MaB and MiB performed the data analysis and interpretation. MiB wrote the first and final draft of the manuscript. MaB is the PI of the project and provided substantial sections of the manuscript. All authors read, revised and approved the final manuscript.

## Pre-publication history

The pre-publication history for this paper can be accessed here:

http://www.biomedcentral.com/1472-6947/14/64/prepub
